# Cytopathological Findings of Secretory Carcinoma of the Salivary Gland and the Diagnostic Utility of Giemsa Staining

**DOI:** 10.3390/diagnostics11122284

**Published:** 2021-12-07

**Authors:** Yuria Egusa, Midori Filiz Nishimura, Satoko Baba, Kengo Takeuchi, Takuma Makino, Tomoyasu Tachibana, Asami Nishikori, Azusa Fujita, Hiroyuki Yanai, Yasuharu Sato

**Affiliations:** 1 Division of Pathophysiology, Okayama University Graduate School of Health Sciences, Okayama 700-8558, Japan; pb4k7y8z@s.okayama-u.ac.jp (Y.E.); asami.kei@s.okayama-u.ac.jp (A.N.); ptqe7laf@s.okayama-u.ac.jp (A.F.); 2 Department of Pathology, Okayama University Graduate School of Medicine, Dentistry and Pharmaceutical Sciences, Okayama 700-8558, Japan; 3 Department of Diagnostic Pathology, Okayama University Hospital, Okayama 700-8558, Japan; yanaih@md.okayama-u.ac.jp; 4 Department of Pathology, The Cancer Institute Hospital, Japanese Foundation for Cancer Research, Tokyo 135-8550, Japan; satoko.baba@jfcr.or.jp (S.B.); kentakeuchi-tky@umin.net (K.T.); 5 Division of Pathology, Cancer Institute, Japanese Foundation for Cancer Research, Tokyo 135-8550, Japan; 6 Pathology Project for Molecular Targets, Cancer Institute, Japanese Foundation for Cancer Research, Tokyo 135-8550, Japan; 7 Department of Otolaryngology, Okayama University Graduate School of Medicine, Dentistry and Pharmaceutical Sciences, Okayama 700-8558, Japan; takmak0617@yahoo.co.jp; 8 Department of Otolaryngology, Japanese Red Cross Society Himeji Hospital, Himeji 670-8540, Japan; tomoyasutachibana@hotmail.co.jp

**Keywords:** secretory carcinoma, salivary gland, mammary analogue secretory carcinoma, Giemsa staining, cytopathology, fine-needle aspiration, *ETV6-NTRK3* fusion

## Abstract

Secretory carcinoma is a salivary gland neoplasm first described as a mammary analogue secretory carcinoma by Skalova and redesignated as a secretory carcinoma in the 2017 World Health Organization Classification of Head and Neck Tumors. Secretory carcinoma diagnosis is reliant on specific cytological and histological findings and the detection of an *ETV6-NTRK3* fusion gene. Here, we examined the clinical and cytopathological features of four cases of secretory carcinoma occurring in three males and a female, aged between 39 and 74 years. All four tumors involved the parotid gland, and were found to have the *ETV6-NTRK3* fusion gene. Fine-needle aspiration-based cytology smears of all tumors displayed papillary and/or dendritic pattern clusters, some of which were associated with blood vessels. The neoplastic cells displayed enlarged nuclei with fine chromatin and small, distinct, single nucleoli. Furthermore, several neoplastic cells with a characteristic vacuolated cytoplasm were identified in each specimen. Giemsa staining revealed cytoplasmic vacuolation, intracytoplasmic metachromatic secretions and/or various sized metachromatic granules, and a background of metachromatic mucin in all four specimens. Given this, we conclude that these cytological findings, especially those of the Giemsa staining, might be helpful in the diagnosis of secretory carcinoma.

## 1. Introduction

In 2010, Skalova et al. described a novel salivary gland neoplasm, a counterpart of secretory carcinoma of the breast, mammary analogue secretory carcinoma (MASC) [1]. In the 2017 World Health Organization Classification of Head and Neck Tumors, MASC was redesignated as a secretory carcinoma [2]. A secretory carcinoma is a low-grade carcinoma that, prior to definition, had been diagnosed as acinic cell carcinoma or other [3]. It usually presents in adults as a slow-growing mass in the parotid, submandibular, or minor salivary glands, and with an equal sex distribution [2]. They typically present with a papillary and microcystic architecture, which is unusual in acinic cell carcinomas. Similarly to secretory carcinoma of the breast, secretory carcinoma harbors a characteristic t(12;15)(p13;q25) mutation that results in an *ETV6-NTRK3* fusion gene [1].

A definitive diagnosis of secretory carcinoma requires confirmation using cytological and histological findings, combined with the detection of the *ETV6-NTRK3* fusion gene by fluorescence in situ hybridization (FISH) or reverse transcription PCR (RT-PCR). However, clinical diagnosis is often made based on cytological and histological findings alone, without searching for the *ETV6-NTRK3* fusion gene. Fine-needle aspiration cytology is widely used as a preoperative diagnostic tool for salivary gland lesions, and is the first test used to diagnose secretory carcinoma. If secretory carcinoma is suspected on the basis of cytology and/or pathology, it is appropriate to recommend performing a test to detect the *ETV6-NTRK3* fusion, which would contribute to the definitive diagnosis of secretory carcinoma at an early stage.

In this report, we investigate the cytological findings of secretory carcinoma by fine-needle aspiration and the utility of Giemsa staining with a literature review. The purpose is to emphasize the necessity of Giemsa staining for the preoperative diagnosis and differential diagnosis of secretory carcinoma.

## 2. Materials and Methods

### 2.1. Case Selection

We searched for major salivary gland tumors diagnosed as acinic cell carcinoma, MASC, or secretory carcinoma from cases diagnosed between 2010 and 2021 at the Department of Pathology at Okayama University (Okayama, Japan). Of these, four cases that underwent fine-needle aspiration cytology were analyzed clinically, cytologically and histologically. Case 2 was reported in the Japanese language [4].

The study protocol was approved by the Institutional Review Board of Okayama University, Okayama, Japan (IRB approval number: 2241) and the study was performed in accordance with the ethical standards outlined in the Declaration of Helsinki. Informed consent was obtained via an opt-out form on the website from three patients and in a written form from one patient (case 1).

### 2.2. Cytological Examination

The fine needle aspiration specimens were smeared onto glass slides, fixed in 95% ethanol for Papanicolaou staining, and air-dried for May–Giemsa staining. These staining slides were made at the same time.

### 2.3. Histological Examination and Immunohistochemistry

Surgically resected specimens were fixed in 10% formalin and embedded in paraffin. Serial sections (3 µm) were obtained from each paraffin-embedded tissue block and stained with hematoxylin and eosin (H&E) and diastase-periodic acid-Schiff (d-PAS).

Immunohistochemical staining was performed on paraffin sections with an automated Ventana Benchmark ULTRA instrument (Roche, Basel, Switzerland) using the following primary antibodies: mammaglobin (304-1A5, 1:2; Agilent, Santa Clara, CA, USA), GCDFP-15 (23A3, ready to use; Agilent), S-100 (polyclonal, ready to use; Agilent), CAM5.2 (CAM5.2, 1:5; BD, Franklin Lakes, NJ, USA), EMA (E29, ready to use; Agilent), p63 (4A4, ready to use; Nichirei, Tokyo, Japan), α-SMA (1A4, 1:50; Agilent), GFAP (EP672Y, ready to use; Roche), and Trypsin (MAB1482, 1:10,000; Chemicon, Tokyo, Japan).

### 2.4. Detection of the ETV6-NTRK3 Fusion Gene

We used FISH or RT-PCR to identify *ETV6-NTRK3* rearrangements in the neoplastic cells from the four specimens.

FISH was performed using probes for *ETV6* and *NTRK3* derived from the bacterial artificial chromosome (BAC) clone (catalog number: 96012 and RPCI11.C; Life Technologies, Carlsbad, CA). The names of the BAC clones used will be provided upon request. The 3 µm sections were deparaffinized in xylene, rehydrated in a 100–85–70% ethanol series, and then washed in phosphate-buffered saline. These specimens underwent a proteolytic treatment, and the tissue sections and probes were then co-denatured at 72 °C for 5 min prior to being hybridized at 37 °C over a period of two nights. Nuclei were counterstained using a DAPI/antifade solution and slides were examined under a fluorescence microscope (BX51; Olympus, Tokyo, Japan). RT-PCR was performed as previously described [5].

## 3. Results

### 3.1. Clinical Findings

The clinicopathological data for four patients are summarized in Table 1. Our cohort consisted of tumors from three male and one female patient aged between 39 and 74 years, with a mean age of 55.3 years. All four cases presented with a parotid gland tumor and were treated surgically with superficial parotidectomy in two cases (cases1 and 3) and total parotidectomy in two cases (cases 2 and 4), but no patient underwent postoperative radiation therapy or chemotherapy. The tumors ranged in size from 1.5 to 3.5 cm, with a mean size of 2.3 cm. Two patients were diagnosed as stage I, one patient was stage III and another patient was stage IVA. Regional lymph node excision was only performed on case 2. No lymph node metastasis was found, however, the patient was classified as pT3N0 due to invasion of the masseter muscle. Case 4, which was stage IVA, was classified as cT4a because of facial paralysis, and pathologically showed invasion into the facial nerve after the tumor resection. The follow-up period ranged from 9 months to 11 years, with a mean follow-up period of 65.5 months. One patient died of primary disease following lung metastasis, while the other three patients were alive and disease-free at the end of their follow-up period.

### 3.2. Cytological Findings

**Figure 1:**
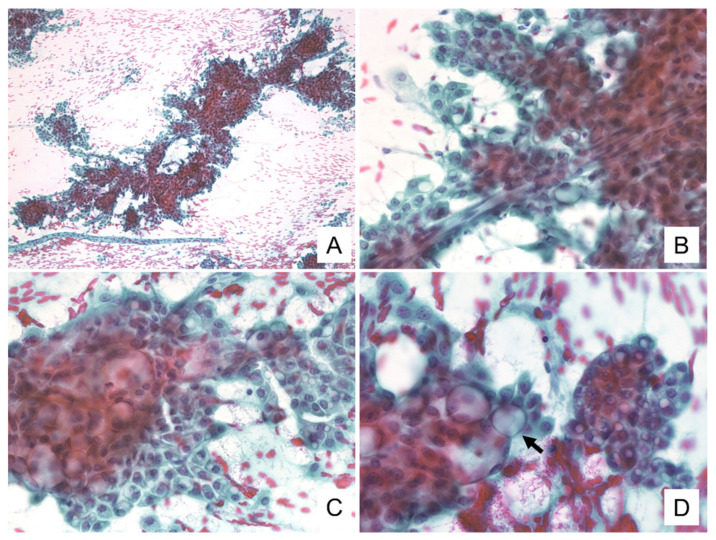
Cytologic findings of Papanicolaou staining. (**A**) Large clusters with papillary to dendritic pattern and hemorrhagic background are visible. (**B**) Blood vessels penetrate into the cell cluster. The neoplastic cells have vacuolated cytoplasm and enlarged nuclei, with fine chromatin and small, distinct single nucleoli. (**C**) The cell borders are distinct. The hyaline globule-like structures are seen. (**D**) Neoplastic cells have numerous cytoplasmic vacuoles, and some of them contain secretions or show intracytoplasmic lumina (allow) ((**A**–**D**): Case 1. (**A**): ×100, (**B**,**C**): ×400, (**D**): ×600).

**Figure 2:**
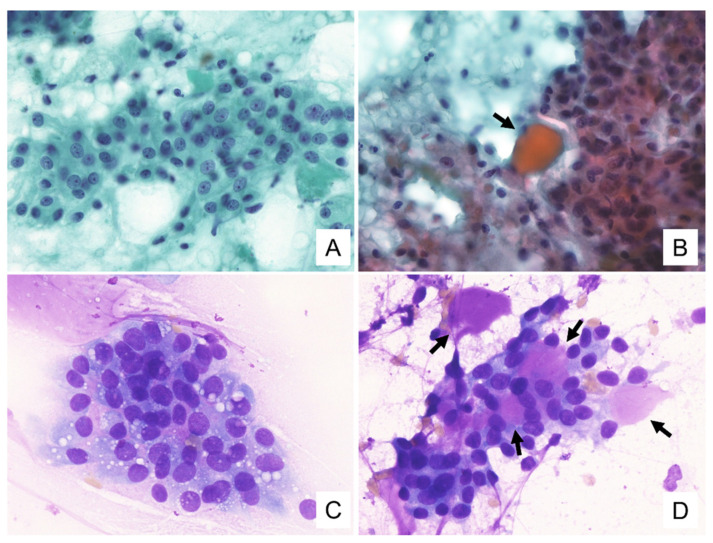
Cytologic findings of case 4. (**A**) Mucinous and cystic backgrounds are visible. Cytoplasmic vacuoles of neoplastic cells are indistinct. (**B**) Mucinous background and cell clusters with hyaline globules are seen (allow). (**C**) Metachromatic mucin in the background. Numerous cytoplasmic vacuoles are visible. (**D**) Metachromatic hyaline globules are visible (allows). ((**A**–**D**): ×600. (**A**,**B**): Papanicolaou staining, (**C**,**D**): Giemsa staining).

**Figure 3:**
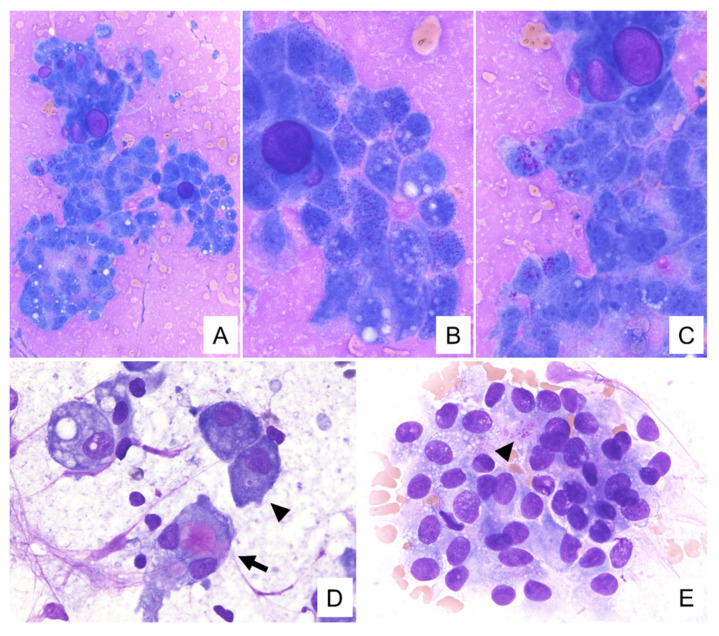
Cytologic findings of Giemsa staining. (**A**) The metachromatic mucin is spread evenly across the background, and metachromatic hyaline globules surrounded by neoplastic cells are visible. (**B**,**C**) Various sized vacuoles and metachromatic granules are in the cytoplasm. (**D**,**E**) Metachromatic secretions ((**D**), allow) and granules ((**D**,**E**), allow heads) are present in the cytoplasm ((**A**–**C**): case 1, (**D**): case 3, (**E**): case 4. (**A**): ×200, (**B**–**E**): ×600).

The cytological features for these tumors are summarized in Table 2. Cytological smears for all four specimens were shown to be cell-rich and demonstrated loosely cohesive clusters. They presented with papillary and/or dendritic patterns, some of which (cases 1, 2, and 4) were associated with blood vessels (Figure 1A,B). In all cases, the neoplastic cells were relatively uniform throughout, and the cell borders were distinct (Figure 1C). These cells had enlarged nuclei with fine chromatin and small, distinct, single nucleoli (Figure 1B,C). In addition, each specimen contained some neoplastic cells with a characteristic vacuolated cytoplasm. These cells were characterized by a single prominent vacuole or multiple small vacuoles (Figure 1B–D). The hyaline globule-like structures were also observed in cases 1, 2, and 4 (Figure 1C and Figure 2B). In case 4, these vacuolated cells were indistinct under the Papanicolaou staining, but could be seen on the Giemsa staining (Figure 2). In all cases, some of these vacuoles contained secretions showing metachromasia when evaluated using Giemsa staining (Figure 3). Occasionally in cases 1 and 2, their secretions contained intracytoplasmic lumina (Figure 1D). In addition to these findings, Giemsa staining showed the metachromatic mucin spread evenly across the background, and the metachromatic hyaline globules surrounded by neoplastic cells (Figure 2D and Figure 3A–C). In case 4, the metachromatic mucin in the background was shown to be stuck to the cell clusters, and numerous foamy macrophages were also observed, suggesting that this tumor was undergoing some form of cystic change. Giemsa staining also revealed numerous small-to-large metachromatic granules in the cytoplasm of the neoplastic cells in all four cases (Figure 3).

### 3.3. Histological and Immunohistochemical Findings

**Figure 4:**
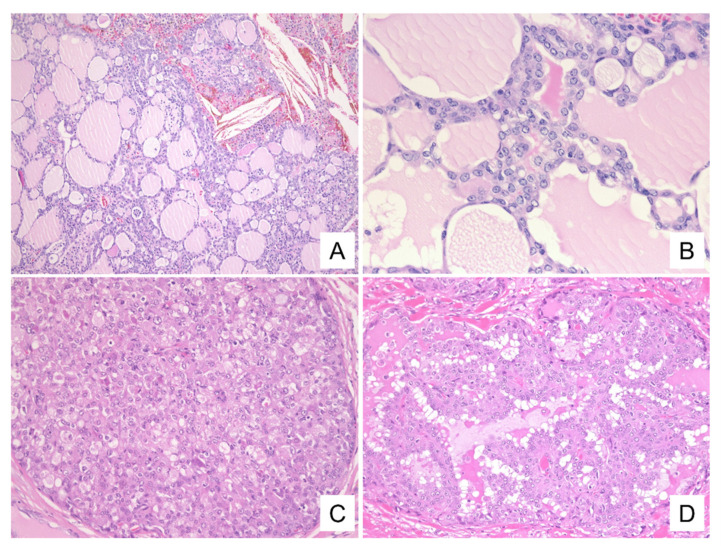
Histologic findings. Histologically, the tumor shows (**A**) follicular-like structures of various sizes resembling thyroid tissue and cystic or microcystic structures. (**B**) The lumens of the follicular structures are filled with eosinophilic, homogeneous secretory materials mimicking the thyroid colloid. The neoplastic cells have vacuolated cytoplasm and enlarged nuclei with small and distinct single nucleoli. In addition, (**C**) microcystic/solid and (**D**) papillary structures are seen ((**A**–**D**): H&E staining. (**A**): case 1, ×40, (**B**): case 1, ×400, (**C**): case 2, ×200, (**D**): case 4, ×400).

**Figure 5:**
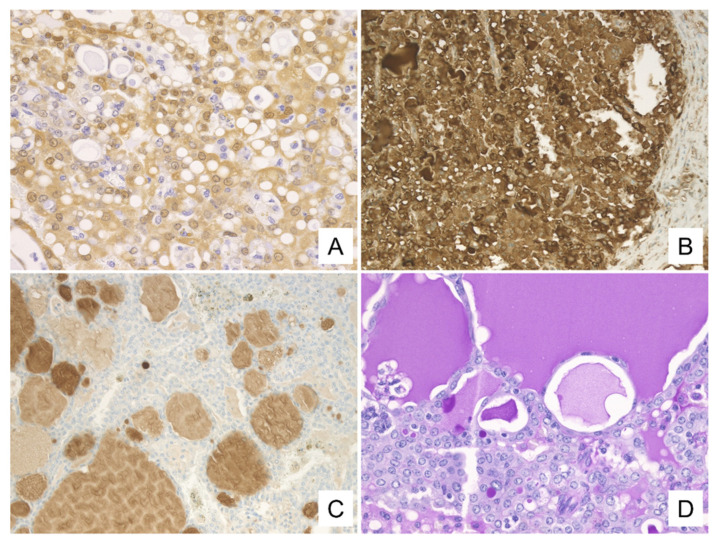
Immunohistochemical findings. Immunohistochemically, neoplastic cells are positive for S-100 (**A**) and mammaglobin (**B**). The secretions are positive for mammaglobin (**B**) and GCDFP-15 (**C**). In addition, d-PAS staining reveals intracytoplasmic and intraluminal secretions and intracytoplasmic granules ((**A**–**C**): case 1, immunohistochemical staining. (**A**): ×400, (**B**,**C**) ×200, (**D**): case 1, d-PAS staining, ×400).

The histological patterns of these tumors are summarized in Table 2. All four presented as solid tumors with follicular-like structures of various sizes resembling thyroid tissue or microcystic/solid structures (Figure 4A,C). The lumens of the follicular structures and the microcysts were filled with eosinophilic, homogeneous secretory materials mimicking the thyroid colloid (Figure 4B). These luminal secretions were positive for d-PAS (Figure 5D). Furthermore, partial papillary structures were observed in cases 1 and 3, and the papillary structures intermingled with the follicular and solid structures in case 4 (Figure 4D). The neoplastic cells, consistent with cytological findings, presented with vacuolated cytoplasm, enlarged nuclei with fine chromatin, and single small nucleoli (Figure 4B).

The immunohistochemical findings are summarized in Table 3. Immunohistochemistry (IHC) showed that all of the neoplastic cells were positive for S-100 and mammaglobin (Figure 5A,B). Also, mammaglobin was positive for the secretions. The secretions of two cases (1 and 2) were positive for GCDFP-15 (Figure 5C). One case was partially positive for GCDFP-15 (case 4), and one was not evaluated for this protein (case 3). In addition, p63 was evaluated in two cases (cases 1 and 4) and was found to be absent in both.

### 3.4. ETV6-NTRK3 Fusion Gene

The *ETV6-NTRK3* fusion was detected in all cases. The fusions in cases 1, 3, and 4 were detected by FISH while the fusion in case 2 was detected by RT-PCR.

## 4. Discussion

Secretory carcinoma is a relatively new class of tumor, first described as MASC in 2010 and later redefined in the WHO classification of Head and Neck Cancers in 2017. This malignancy is characterized by the presence of an *ETV6-NTRK3* fusion. Previous reports classified secretory carcinoma as low-grade adenocarcinoma, low-grade mucoepidermoid carcinoma, or acinic cell carcinoma of the salivary gland [3]. In fact, case 1 was diagnosed as a follicular variant of acinic cell carcinoma. Therefore, the frequency of secretory cancers is not well defined, and they may be more frequent than currently reported. It can still be difficult to successfully identify these tumors without an *ETV6-NTRK3* fusion gene result. Recently, anti-tropomyosin receptor kinase (TRK) inhibitors have been used as a new therapy for cancers with *NTRK* fusion genes, such as secretory carcinoma. Therefore, it has become even more important to differentiate secretory carcinoma with the *ETV6-NTRK3* fusion gene from other salivary gland tumors.

The clinicopathological features of our cases included a mean age of 55.3 years (range: 39–74 years) and a male-to-female ratio of 3:1. All four tumors were isolated from the parotid gland. These data are compatible with the characteristics of these tumors from other studies [1,6,7,8]. The incidence of secretory carcinoma in all parotid gland carcinomas was 4.5% during the period 2013 to 2020 at our university. Secretory carcinoma usually presents with an indolent clinical course. Lymph node metastases are reported in approximately 25% of patients, but distant metastases are rare [2]. One of our cases (case 2) experienced metastasis to the lung without lymph node metastasis, leading to death from the disease. There was also a case that showed invasion into the facial nerve (case 4). These results suggest that secretory carcinoma may be slightly more aggressive than acinic cell carcinoma [3,9,10,11,12].

Several studies on the cytological features of secretory carcinoma [5,7,13,14,15,16] have described some common features of these tumors, including loosely cohesive epithelial clusters, round nuclei with powdery chromatin and small nucleoli, and somewhat vacuolated cytoplasm without distinct zymogen granules. Furthermore, some reports have indicated that a mucinous background is one of the cytological characteristics of secretory carcinoma [5].

Our cases shared the common features mentioned above, and papillary patterns, vacuolated cytoplasm, nuclei with fine chromatin and distinct nucleoli, and a mucinous background showing metachromasia on Giemsa staining, were observed in all cases. In addition, metachromatic granules of various sizes were observed in the cytoplasm in all four cases, and neoplastic cells with intracytoplasmic metachromatic secretions and metachromatic hyaline globules surrounded by neoplastic cells were also observed in three cases (case 1,2,3). In case 4, cytoplasmic vacuolation was indistinct on the Papanicolaou staining, and pleomorphic adenoma was suspected based on these cytological findings. However, when reviewed in the present study, cytoplasmic vacuolation was clearly detected by Giemsa staining. Based on these results, we believe that the background metachromatic mucin and intracytoplasmic metachromatic granules on Giemsa staining could be used as cytological values for the cytology-based diagnosis of secretory carcinoma. Furthermore, we suggest that Giemsa staining can capture the cytoplasmic vacuolation characteristic of secretory carcinoma more clearly than Papanicolaou staining.

The background mucin, intracytoplasmic secretions and granules, and hyaline globules were similarly metachromatic on the Giemsa staining, suggesting that they may have the same composition. In addition, both large and small metachromatic granules were found in the cytoplasm. This suggests that they were part of the secretions produced by the neoplastic cells, which aggregated in the cytoplasm, gradually became larger, and were eventually secreted out of the cells. It is thought that these appeared as background mucin and hyaline globules. Histologically, the intracytoplasmic and intraluminal secretions were positive in d-PAS staining, suggesting that they also have the same composition. Therefore, the hyaline globules, on cytology, may correspond to the intraluminal secretions seen on histology.

Previous reports have also described metachromatic extracellular material and intracytoplasmic granules, and clear or metachromatic cytoplasmic vacuoles following Giemsa staining of secretory carcinoma [7,16,17,18,19]. Among the various low-grade salivary gland tumors, pleomorphic adenoma, Warthin tumor with mucinous metaplasia, low-grade mucoepidermoid carcinoma, and secretory carcinomas are best known for producing extracellular mucinous material, which is best identified using modified Giemsa staining. Levine et al. [7] reported that secretory carcinoma are characterized by a web-like metachromatic extracellular material, which is quite different from the thick mucin (blue on Giemsa staining) observed in Warthin tumors and low-grade mucoepidermoid carcinomas, and also different from the characteristic fibrillary matrix of pleomorphic adenoma. In addition, neoplastic cells from acinic cell carcinoma and low-grade cribriform cystadenocarcinoma have been reported to exhibit metachromatic cytoplasmic vacuoles and/or intracytoplasmic granules similar to those found in secretory carcinoma. However, conditions characterized by neoplastic lesions with metachromatic cytoplasmic vacuoles and metachromatic intracytoplasmic granules are most likely to be secretory carcinoma. In the future, we suggest that such findings mediated by Giemsa staining should be treated as likely to be secretory carcinoma and should be submitted to testing for the presence of the *ETV6-NTRK3* fusion gene.

In this report, only one case (case 3) was diagnosed as MASC or secretory carcinoma at the original FNAC diagnosis (Table 1). Case 3 was able to be diagnosed as secretory carcinoma from the cytology, based on the presence of vacuolated cytoplasm, intracytoplasmic secretions and mammaglobin-positive findings by cell block, which are characteristic of secretory carcinoma. Case 1 was diagnosed in 2010, when the classification and definition of secretory carcinoma had not yet been established. Therefore, despite the characteristic findings of secretory carcinoma on cytology, the patient was not diagnosed with secretory carcinoma. Now that the definition of secretory carcinoma has been established, we believe that this case could have been reliably diagnosed as secretory carcinoma. In cases 2 and 4, characteristic findings were observed on cytology, but the cytological diagnosis was incorrect. Since secretory carcinoma is a relatively new classification, it is possible that the cytologists and pathologists did not understand the correct cytological findings of secretory carcinoma. We hope that this report will help reinforce the need to absorb new knowledge and incorporate the findings into everyday practice.

Fine-needle aspiration cytology is a well-accepted technique for the evaluation of tumors in the head and neck region, as it is less invasive and can be easily applied for the characterization of tumors using ancillary techniques such as cytogenetics, cell block immunohistochemistry, and electron microscopy [20,21,22,23,24]. Giemsa staining is one of the most common clinical staining methods in cytology. To the best of our knowledge, there are no previous reports detailing the usefulness of Giemsa staining in the fine-needle aspiration cytology of secretory carcinoma. We suggest that Giemsa staining combined with Papanicolaou staining may be a useful tool for the cytological diagnosis of secretory carcinoma, as shown in case 4. In addition, the molecular detection of the *ETV6-NTRK3* fusion, which is currently used for the definitive diagnosis of secretory carcinoma, is expensive and not routinely performed in many hospitals. On the other hand, Giemsa staining is a low-cost method that is routinely used in most hospitals. If a high likelihood of secretory carcinoma can be determined by Giemsa staining, it will be easier to reliably diagnose secretory carcinoma.

There have been reports describing the presence of salivary gland tumors that are positive for S-100 and mammaglobin but without the *ETV6-NTRK3* fusion, making them similar, but not identical to, secretory carcinoma [25]. Moreover, t(12;15)(p13;q25) leading to an *ETV6-NTRK3* fusion is a characteristic chromosomal translocation associated with cancers in all of the germ layers, including secretory breast carcinoma, congenital fibrosarcoma, congenital mesoblastic nephroma, and acute myelogenous leukemia [26,27,28,29,30,31]. Therefore, it is difficult to identify the cellular origins of secretory carcinoma. S-100 immunostaining is positive in secretory carcinoma, and S-100-positive cells include centroacinar cells, ductal cells, and pancreatic nerve cells, which are similar in morphology and function to salivary glands [32]. This leads us to consider that the cells of the salivary gland, which are similar to the centroacinar cells of the pancreas, are likely the cellular origin for secretory carcinoma of the salivary gland, but further substantiation of this hypothesis is required.

In conclusion, we describe here the usefulness of Giemsa staining in the cytological diagnosis of secretory carcinoma. The cytological findings of background metachromatic mucin, cytoplasmic vacuolation, and intracytoplasmic metachromatic secretions and/or granules of various sizes on Giemsa staining are likely to be helpful in the cytological diagnosis of secretory carcinoma.

## Figures and Tables

**Table 1:** Clinicopathological features of secretory carcinoma.

Case No.	Age/Sex	Location	Size (cm)	TNM	Stage	Original FNAC Diagnosis	Follow-Up
1	39/F	Left parotid gland	1.8	cT1N0M0 pT1	cStage I pStage I	Indeterminate/neoplastic lesion	NED, 11 years
2	61/M	Left parotid gland	3.0	cT2N0M0 pT3N0	cStage II pStage III	Carcinoma	DOD, 7 years
3	47/M	Right parotid gland	1.5	cT1N0M0 pT1	cStage I pStage I	MASC	NED, 3 years
4	74/M	Right parotid gland	2.8	cT4aN0M0 pT4a	cStage IVA pStage IVA	Pleomorphic adenoma	NED, 9 months

FNAC; fine needle aspiration cytology, MASC; mammary analogue secretory carcinoma, DOD; died of disease, NED; no evidence of diseases.

**Table 2:** Histological and cytological findings of secretory carcinoma.

	Case 1	Case 2	Case 3	Case 4
**Histologic findings**	Follicular/Microcystic/Solid/partially Papillary	Follicular/Microcystic/Solid	Solid/Microcystic/partially Papillary	Follicular/Papillary/Solid
**Cytologic findings**				
Cellularity	High	High	High	High
Cluster patterns	Papillary and dendritic structures with transgressing vessels/tubular gland structure with metachromatic hyaline globules	Papillary and dendritic clusters with transgressing vessels/tubular gland structure with metachromatic hyaline globules	Papillary clusters/tubular gland structure	Papillary clusters with transgressing vessels/tubular gland structure with metachromatic hyaline globules
Nuclei features	Round to oval; fine chromatin; prominent nucleoli	Round to oval; fine chromatin; prominent nucleoli	Round to oval; fine chromatin; prominent nucleoli	Round to oval; fine chromatin; prominent nucleoli
Papanicolaou staining background	Hemorrhagic, mucinous and cystic	Cystic	Mucinous and cystic	Mucinous and cystic
Cytoplasmic features	Variously-sized vacuoles, occasionally with ICL	Variously-sized vacuoles occasionally with ICL	Variously-sized vacuoles	Indistinct vacuoles
Giemsa staining background	Metachromatic mucin	Metachromatic mucin	Metachromatic mucin	Metachromatic mucin
Cytoplasmic features	Variously-sized vacuoles, metachromatic secretions and granules	Variously-sized vacuoles, metachromatic secretions and granules	Variously-sized vacuoles, metachromatic secretions and granules	Variously-sized vacuoles, metachromatic secretions and granules

ICL; intracytoplasmic lumina.

**Table 3:** Summary of immunohistochemical features.

	Case 1	Case 2	Case 3	Case 4
S-100	+	+	+	+
mammaglobin	+	+	+	partially +
GCDFP-15	+(Secretion)	+(Secretion)	ND	partially +
CAM5.2	+	ND	ND	ND
EMA	partially +	ND	ND	ND
p63	-	ND	ND	ND
α-SMA	-	ND	ND	ND
GFAP	-	ND	ND	-

ND; Not done.

## Data Availability

Not applicable.

## References

[1] Skálová A., Vanecek T., Sima R., Laco J., Weinreb I., Perez-Ordonez B., Starek I., Geierova M., Simpson R.H., Passador-Santos F. (2010). Mammary analogue secretory carcinoma of salivary glands, containing the ETV6-NTRK3 fusion gene: A hitherto undescribed salivary gland tumor entity. Am. J. Surg. Pathol..

[2] WHO (2017). WHO Classification of Head and Neck Tumors.

[3] Chiosea S.I., Griffith C., Assaad A., Seethala R.R. (2012). Clinicopathological characterization of mammary analogue secretory carcinoma of salivary glands. Histopathology.

[4] Nasu A., Hata S., Fjita M., Yamauchi T., Nakamura S., Tanaka K., Ichimura K., Yanai H. (2016). Mammary analogue secretory carcinoma of parotid gland—A case report. J. Jpn. Soc. Clin. Cytol..

[5] Higuchi K., Urano M., Takahashi R.H., Oshiro H., Matsubayashi J., Nagai T., Obikane H., Shimojo H., Nagao T. (2014). Cytological features of mammary analogue secretory carcinoma of salivary gland: Fine-needle aspiration of seven cases. Diagn. Cytopathol..

[6] Bishop J.A., Yonescu R., Batista D., Eisele D.W., Westra W.H. (2013). Most nonparotid “acinic cell carcinomas” represent mammary analog secretory carcinomas. Am. J. Surg. Pathol..

[7] Levine P., Fried K., Krevitt L.D., Wang B., Wenig B.M. (2014). Aspiration biopsy of mammary analogue secretory carcinoma of accessory parotid gland: Another diagnostic dilemma in matrix-containing tumors of the salivary glands. Diagn. Cytopathol..

[8] Khalele B.A. (2017). Systematic review of mammary analog secretory carcinoma of salivary glands at 7 years after description. Head Neck.

[9] Jung M.J., Song J.S., Kim S.Y., Nam S.Y., Roh J.L., Choi S.H., Kim S.B., Cho K.J. (2013). Finding and characterizing mammary analogue secretory carcinoma of the salivary gland. Korean J. Pathol..

[10] Jung M.J., Kim S.Y., Nam S.Y., Roh J.L., Choi S.H., Lee J.H., Baek J.H., Cho K.J. (2015). Aspiration cytology of mammary analogue secretory carcinoma of the salivary gland. Diagn. Cytopathol..

[11] Sethi R., Kozin E., Remenschneider A., Meier J., VanderLaan P., Faquin W., Deschler D., Frankenthaler R. (2014). Mammary analogue secretory carcinoma: Update on a new diagnosis of salivary gland malignancy. Laryngoscope.

[12] Skálová A., Vanecek T., Majewska H., Laco J., Grossmann P., Simpson R.H., Hauer L., Andrle P., Hosticka L., Branžovský J. (2014). Mammary analogue secretory carcinoma of salivary glands with high-grade transformation: Report of 3 cases with the ETV6-NTRK3 gene fusion and analysis of TP53, β-catenin, EGFR, and CCND1 genes. Am. J. Surg. Pathol..

[13] Pisharodi L. (2013). Mammary analog secretory carcinoma of salivary gland: Cytologic diagnosis and differential diagnosis of an unreported entity. Diagn. Cytopathol..

[14] Bishop J.A., Yonescu R., Batista D.A., Westra W.H., Ali S.Z. (2013). Cytopathologic features of mammary analogue secretory carcinoma. Cancer Cytopathol..

[15] Petersson F., Lian D., Chau Y.P., Yan B. (2012). Mammary analogue secretory carcinoma: The first submandibular case reported including findings on fine needle aspiration cytology. Head Neck Pathol..

[16] Griffith C.C., Stelow E.B., Saqi A., Khalbuss W.E., Schneider F., Chiosea S.I., Seethala R.R. (2013). The cytological features of mammary analogue secretory carcinoma: A series of 6 molecularly confirmed cases. Cancer Cytopathol..

[17] Takeda M., Kasai T., Morita K., Takeuchi M., Nishikawa T., Yamashita A., Mikami S., Hosoi H., Ohbayashi C. (2015). Cytopathological features of mammary analogue secretory carcinoma—Review of literature. Diagn. Cytopathol..

[18] Oza N., Sanghvi K., Shet T., Patil A., Menon S., Ramadwar M., Kane S. (2016). Mammary analogue secretory carcinoma of parotid: Is preoperative cytological diagnosis possible?. Diagn. Cytopathol..

[19] Rodríguez-Urrego P.A., Dogan S., Lin O. (2017). Cytologic findings of mammary analogue secretory carcinoma arising in the thyroid. Diagn. Cytopathol..

[20] Sergi C., Dhiman A., Gray J.A. (2018). Fine Needle Aspiration Cytology for Neck Masses in Childhood. An Illustrative Approach. Diagnostics.

[21] Jain M., Majumdar D.D., Agarwal K., Bais A.S., Choudhury M. (1999). FNAC as a diagnostic tool in pediatric head and neck lesions. Indian Pediatr..

[22] Mittra P., Bharti R., Pandey M.K. (2013). Role of fine needle aspiration cytology in head and neck lesions of paediatric age group. J. Clin. Diagn. Res..

[23] Rapkiewicz A., Thuy Le B., Simsir A., Cangiarella J., Levine P. (2007). Spectrum of head and neck lesions diagnosed by fine-needle aspiration cytology in the pediatric population. Cancer.

[24] Handa U., Mohan H., Bal A. (2003). Role of fine needle aspiration cytology in evaluation of paediatric lymphadenopathy. Cytopathology.

[25] Patel K.R., Solomon I.H., El-Mofty S.K., Lewis J.S., Chernock R.D. (2013). Mammaglobin and S-100 immunoreactivity in salivary gland carcinomas other than mammary analogue secretory carcinoma. Hum. Pathol..

[26] Li Z., Tognon C.E., Godinho F.J., Yasaitis L., Hock H., Herschkowitz J.I., Lannon C.L., Cho E., Kim S.J., Bronson R.T. (2007). ETV6-NTRK3 fusion oncogene initiates breast cancer from committed mammary progenitors via activation of AP1 complex. Cancer Cell.

[27] Tognon C., Knezevich S.R., Huntsman D., Roskelley C.D., Melnyk N., Mathers J.A., Becker L., Carneiro F., MacPherson N., Horsman D. (2002). Expression of the ETV6-NTRK3 gene fusion as a primary event in human secretory breast carcinoma. Cancer Cell.

[28] Knezevich S.R., McFadden D.E., Tao W., Lim J.F., Sorensen P.H. (1998). A novel ETV6-NTRK3 gene fusion in congenital fibrosarcoma. Nat. Genet..

[29] Knezevich S.R., Garnett M.J., Pysher T.J., Beckwith J.B., Grundy P.E., Sorensen P.H. (1998). ETV6-NTRK3 gene fusions and trisomy 11 establish a histogenetic link between mesoblastic nephroma and congenital fibrosarcoma. Cancer Res..

[30] Rubin B.P., Chen C.J., Morgan T.W., Xiao S., Grier H.E., Kozakewich H.P., Perez-Atayde A.R., Fletcher J.A. (1998). Congenital mesoblastic nephroma t(12; 15) is associated with ETV6-NTRK3 gene fusion: Cytogenetic and molecular relationship to congenital (infantile) fibrosarcoma. Am. J. Pathol..

[31] Eguchi M., Eguchi-Ishimae M., Tojo A., Morishita K., Suzuki K., Sato Y., Kudoh S., Tanaka K., Setoyama M., Nagamura F. (1999). Fusion of ETV6 to neurotrophin-3 receptor TRKC in acute myeloid leukemia with t(12; 15)(p13; q25). Blood.

[32] Haimoto H., Hosoda K., Kato K. (1987). Differential distribution of immunoreactive S100-alpha and S100-beta proteins in normal nonnervous human tissues. Lab. Investig..

